# Examining the status quo and related factors of clinical nurses’ spare-time planning

**DOI:** 10.3389/fpubh.2026.1798726

**Published:** 2026-05-12

**Authors:** Nana Liang, Xiaoyan Wang, Jishun Ren, Yajun Zhang, Xiaohui Liu, Lihong Ka, Juan Zhao, HongXia Tao

**Affiliations:** 1 School of Nursing, Gansu Medical College, Pingliang, China; 2 School of Nursing, Hexi University, Zhangye, China; 3 Affiliated Hospital of Gansu Medical College, Pingliang, China; 4 School of Nursing, Ningxia Medical University, Yinchuan, China

**Keywords:** clinical nurses, occupational stress, positive psychological capital, related factors, spare-time planning

## Abstract

**Background:**

Nursing is an important part of healthcare. This study aimed to investigate the current situation of clinical nurses’ spare-time planning and analyse factors associated with this aspect to provide a reference for improving their leisure-time planning ability.

**Methods:**

The study was conducted from May to July 2024, with 827 clinical nurses from four tertiary hospitals in Gansu province, Participants were recruited using convenience sampling method. The Leisure Crafting Scale, Chinese Nurses Stressor Scale, and the Positive Psychological Capital Questionnaire were employed in the analysis.

**Results:**

The average score of spare time planning was (26.84 ± 7.80). Linear regression analysis showed that childbearing status, frequency of weekly sports participation, positive psychological capital, and occupational stress were factors associated with clinical nurses’ spare time planning (*p* < 0.05).

**Conclusion:**

The findings revealed that nurses’ ability to plan their spare time was moderate and needs to be further improved. Nursing managers should rationally arrange nurses’ human resources, guide department members to actively participate in spare-time activities, build harmonious interpersonal relationships, and improve nurses’ spare-time planning.

## Background

Nursing is an important part of healthcare; it is significant for comprehensively promoting the implementation of the Healthy China strategy and actively responding to population ageing (1). During the “14th Five-Year Plan” period, the comprehensive promotion of Healthy China put forward new requirements for the development of nursing. Nursing needs to closely focus on the health needs of the people; build a comprehensive, whole-process, high-quality, and efficient nursing service system; and continuously meet the differentiated nursing service needs of the masses (2). Nursing workers’ concerns are no longer a single reward mechanism. It is also necessary to pay attention to nurses’ psychological changes and needs, to foster their personal initiative and enhance their work motivation (3). Research has found that nurses must maintain interpersonal relationships, balance work and family, and pursue personal development (4). According to the job demands-resources model (5), job demands damage an individual’s health and well-being, especially in a state of high work intensity and low freedom. Therefore, it is necessary to maintain personal resource investments.

Spare-time planning refers to a series of amateur leisure activities that individuals actively perform according to goals, specific interpersonal relationships, and behaviours related to personal development and learning (6). Research has found that spare time-planning plays an important regulatory role in high-intensity work pressure and low-freedom life situations (7). Some studies have shown that spare time planning can reduce negative emotions, offset emotional exhaustion, stimulate positive emotions, and improve individuals’ work efficiency, therefore, playing a crucial role in maintaining a positive work state (8). As a leisure activity, spare-time planning enables nurses to reconstruct their identity cognition and explore their sense of self-meaning. Simultaneously, it helps meet the needs of nurses and improve the quality of nursing services (9–11).

Current research on spare-time planning in the nursing field is still in its initial stages, and discussions on the level of nurses’ spare-time planning are insufficient. The Psychological Capital Theory (12) and the Job Demand-Resources Model (5) indicate that positive psychological capital and occupational stress affect employees’ work performance and personal well-being, thereby shaping employees’ spare-time planning behaviour. Based on this, this study aims to address the following research questions: What is the current status of clinical nurses’ spare-time planning and factors related to work stress? How do positive psychological capital and occupational stress correlate with nurses’ spare-time planning?

## Methods

### Study design

A quantitative design based on a cross-sectional study was used.

### Participants

From May to July 2024, clinical nurses from four tertiary hospitals in Pingliang City, Baiyin City, Wuwei City, and Qingyang City in Gansu Province were selected as survey participants, using convenience sampling. The inclusion criteria were: 1) Registered nurses on clinical duty, and 2) clinical nursing experience of 6 months or more. The exclusion criteria were: 1) Individuals studying or interning in the hospital, and 2) Head nurses or those in other managerial positions. All the participants provided written informed consent.

### Ethics approval and consent to participate

This study was approved by the Ethics Committee for Medical Research at Gansu Medical College (1). All the experiments were performed in accordance with the relevant guidelines and regulations of the Declaration of Helsinki.

## Instruments

### Demographic questionnaire

Demographic and occupational characteristics were collected using a self-designed questionnaire. The variables included in this study were: gender, age, educational level, marital status, only-child status, childbearing (reproductive) status, professional title, department, and frequency of sports participation per week. Specifically, the following key variables were measured with single items: Marital status was assessed by asking participants “What is your marital status?” and it included 4 response options: unmarried, married, divorced or widowed, and remarried. Reproductive status was measured through the item “How many children have you given birth to?” with response options of 0 (not given birth), 1 child, and 2 or more children. Only child status was determined by the question “Are you an only child?” with response options—yes or no. Frequency of weekly sports participation was assessed by the item “How often do you participate in sports activities per week?” and response options included 0 times, 1–3 times, and ≥4 times. Other demographic and occupational variables (e.g., age, educational level, professional title, department) were collected using standard single-choice questions.

### Leisure crafting scale (LCS)

The Leisure Crafting Scale was adapted to the Chinese context by Guo et al. (13) to assess nurses’ ability to plan their leisure time. This version consists of nine items rated on a 5-point Likert scale, ranging from 1 (“completely inconsistent”) to 5 (“completely consistent”). Total scores range from 9 to 45, with higher scores indicating greater spare-time planning capability. According to the original classification, scores of 9–22, 23–29, and 30–45 represent low, moderate, and high levels of planning ability, respectively. In this study, spare time planning was measured using this scale. Although originally developed to assess leisure crafting, the scale’s conceptual content aligns closely with the construct of spare time planning. Specifically, it captures individuals’ proactive efforts to organize and structure their leisure time—such as setting goals, seeking new experiences, and pursuing personal growth—all of which reflect intentional planning behaviours. For instance, Item 5 (“I try to set new goals for myself to achieve through leisure activities”) and Item 7 (“Through my leisure activities, I try to obtain novel experiences”) directly tap into the planning and proactive arrangement of leisure time. Furthermore, this scale has been previously validated in a Chinese nursing population, and demonstrated good reliability in the current study (Cronbach’s *α* = 0.918). Therefore, it is considered a suitable instrument for measuring spare time planning in this study.

### Chinese nurses stressor scale (CNSS)

The Chinese Nurses’ Stressor Scale (CNSS) revised by Li et al. (14) was employed to evaluate the status of work-related pressure and its stressors among nursing staff. This scale consists of 35 items categorised into 5 dimensions: nursing profession and work (7 items), workload and time allocation (5 items), work environment and resources (3 items), patient care (11 items), and management and interpersonal relationships (9 items). A 4-level scoring system was employed, with scores ranging from 1 to 4, reflecting a spectrum from ‘strongly disagree’ to ‘strongly agree’. The total score ranges from 35 to 140, with higher scores indicating higher levels of occupational stress. The Cronbach’s *α* coefficient for this scale in the present study was 0.948.

### Positive psychological capital questionnaire (PPCQ)

The Positive Psychological Capital Questionnaire (PPCQ) was developed by Zhang et al. (15) and encompasses 26 items across 4 dimensions: self-efficacy (7 items), resilience (7 items), hope (6 items), and optimism (6 items). The scale employs a 7-point Likert scoring method, with scores ranging from 1 (“completely inconsistent”) to 7 (“completely consistent”). Items 8, 10, 12, 14, and 25 are reverse scored. The total score ranges from 26 to 182, with higher scores indicating a greater level of psychological capital. The Cronbach’s *α* coefficient for this scale in the present study was 0.871.

### Design and quality control

The questionnaires were imported into the online platform WenJuanXing^1^. After the authors obtained consent from the management of each hospital, the head nurses sent the questionnaire link to WeChat groups in the clinical departments. Nurses voluntarily participated in the data collection and completed the electronic questionnaires anonymously. The researchers provided unified instructions at the beginning of the questionnaire to inform participants about the purpose of the investigation and important considerations for completing the questionnaire. A total of 867 questionnaires were collected. After a review by two researchers, those with more than 10% blank responses or evidently patterned answers were excluded. Ultimately, 827 valid questionnaires were retained, resulting in a response rate of 95.4%.

### Data analysis method

Data entry was performed using Excel, and statistical analyses and descriptions were performed using SPSS 25.0. Correlation analyses were performed using Pearson’s method. For single factors, *t*-tests and analysis of variance were used. For multivariate analysis, hierarchical linear regression was employed to identify predictors. Prior to conducting a multivariate regression analysis, multicollinearity among independent variables was assessed using variance inflation factor (VIF), with VIF < 5 indicating no serious multicollinearity. Residual diagnostics were performed to verify the assumptions of normality and homoscedasticity. Specifically, the normal P–P plot of standardized residuals was used to examine normality, and the scatterplot of standardized predicted values versus standardized residuals was used to assess homoscedasticity. Regarding missing data, there were no missing values in this study. All questionnaires were checked for completeness upon collection. Questionnaires with incomplete and invalid questionnaires (e.g., patterned responses) were excluded prior to analysis, resulting in a final sample of 827 valid responses with complete data for all variables. A two-tailed *p* < 0.05 was considered statistically significant.

### Sample size estimate

The sample size was estimated using G*Power 3.1 software, where the required sample size was calculated using F tests and linear multiple regression (fixed model, *R^2^* deviation from zero as the statistical test). A medium effect size of 0.15 was selected, with an alpha level (*α*) of 0.05 for two-sided testing and a desired power of 0.80. This calculation suggests an expected sample size of 68 participants. An additional 20% was added to account for potential attrition, resulting in a target sample size of 82 participants. Finally, the study collected 827 valid cases, exceeding the required sample size.

## Results

### Demographic characteristics and univariable analysis

This study utilized the responses of 827 nurses (11 males and 816 females) for the analysis. The age range of the participants was 20 to 55 years, with a mean age of 33.41 ± 6.53 years. Regarding marital status, 147 participants were unmarried, 669 were married (including those who were remarried), and 11 were divorced or widowed. Regarding employment methods, 210 nurses were formally employed and 617 were engaged in non-formal employment. The distribution of nurses across departments included 329 in internal medicine, 101 in surgery, 27 in the operating room, 79 in the emergency department/intensive care unit, 68 in obstetrics and gynaecology, 73 in paediatrics, 74 in the outpatient department, and 76 in other departments (e.g., oncology, blood purification centres, otolaryngology, and traditional Chinese medicine).

### Clinical nurses’ spare-time planning

Table 1 presents the total score and item scores for clinical nurses’ spare-time planning.

**Table 1:** Total score and the score of each item in shaping clinical nurses’ spare-time planning (*n* = 827, $\bar{x}$ ±s).

Item	Scores
Score for non-working hours	26.84 ± 7.80
Scores of each item during spare time planning	
1. I try to build relationships through leisure activities.	2.75 ± 1.14
2. I try to find challenging activities outside of work.	2.50 ± 1.12
3. I try to increase my skills through leisure activities.	3.07 ± 1.11
4. I try to increase my learning experiences through leisure activities.	3.23 ± 1.09
5. I try to set new goals for myself to achieve through leisure activities.	3.15 ± 1.10
6. Through my leisure activities, I look for inspiration from others.	2.89 ± 1.12
7. Through my leisure activities, I try to obtain novel experiences.	3.11 ± 1.13
8. My leisure time is a chance for me to grow and develop.	3.01 ± 1.12
9. I seek new experiences through leisure activities to keep myself mentally stimulated.	3.13 ± 1.11

### Correlative analysis of spare-time planning, occupational stress, and positive psychological capital of clinical nurses

A correlative analysis was conducted on the three variables of spare-time *planning*, occupational stress, and positive psychological capital among clinical nurses. The results showed that the variables were significantly correlated (*p* < 0.01; Table 2).

**Table 2:** Descriptive statistics and correlation analysis of spare-time planning, occupational stress, and positive psychological capital among clinical nurses (*n* = 827, *r*).

Item	Spare-time planning	Occupational stress	Positive psychological capital
spare time planning	1		
Occupational stress	−0.234***	1	
Positive psychological capital	0.388***	−0.279***	1

***p is less than 0.01.

### Results of univariate analysis of the planning of clinical nurses in their spare time

There was no statistically significant difference in nurses’ spare-time planning scores among different genders, ages, marital statuses, employment methods, or departments (all *p* < 0.05). The items with statistically significant differences are listed in Table 3. It should be noted that in this sample, marital status and reproductive status were highly correlated: all unmarried nurses had not given birth, and all married nurses had given birth. Although marital status was not significantly associated with spare-time planning in the univariate analysis (*p* > 0.05), reproductive status showed a significant association (*p* < 0.05). Due to the high overlap between these two variables and the non-significant finding for marital status, only reproductive status was included as an independent variable in the subsequent multivariate regression analysis, in accordance with standard statistical practice. The variables that showed significance in the univariate analysis (including only-child status, reproductive status, professional title, nursing seniority, frequency of weekly sports participation, number of night shifts per month, positive psychological capital, and occupational stress) were entered into the multivariate linear regression model as independent variables (see Table 3).

**Table 3:** Univariate analysis of the planning of clinical nurses during their spare-time planning (*n* = 827, $\bar{x}$ ±s).

Project		*n*	Spare time planning score	*t/F*	*p*
Whether one is an only child	Yes	52	28.96 ± 7.89	2.031	0.043
	No	775	26.70 ± 7.78		
Reproductive status	Not given birth	227	28.33 ± 7.93	7.172	0.001
	Given birth to 1 child	251	26.88 ± 7.96		
	Given birth to 2 or more children	349	25.84 ± 7.45		
Professional title	Nurse	23	31.74 ± 8.63	3.714	0.011
	Enrolled nurse	539	26.83 ± 7.92		
	Supervisor nurse	210	26.16 ± 7.57		
	Deputy chief nurse and above	55	27.47 ± 6.35		
Nursing seniority (year)	<5	225	28.33 ± 8.20	3.053	0.016
	5 ~ <10	193	26.19 ± 7.68		
	10 ~ <15	230	26.05 ± 7.88		
	15 ~ <20	80	26.89 ± 7.23		
	≥20	99	26.54 ± 6.98		
Frequency of participating in sports every week*	Zero times	463	24.98 ± 7.88	32.205	<0.001
	One to three times	328	29.19 ± 6.95		
	Greater than or equal to 4 times	36	29.39 ± 7.81		
	Zero times	195	26.87 ± 7.63	4.812	0.008
Number of days	1 ~ 3 times	220	28.14 ± 8.17		
	≥4 times	412	26.13 ± 7.59		

^*^Based on the standard of 20 to 30 min each time.

### Multicollinearity diagnostics and residual analysis

Before conducting the multivariate linear regression analysis, multicollinearity among the independent variables was assessed using variance inflation factor (VIF). The results showed that the VIF values for all independent variables ranged from 1.092 to 2.678, all below the recommended cutoff of 5, indicating that multicollinearity was not a serious concern in this study. The standardized residuals in this study ranged from −3.224 to 3.247 (*M* = 0, *SD* = 0.990), with no absolute values exceeding 3.5, indicating the absence of influential outliers. The distribution of the residuals approximated normality, and the assumptions of linearity and homoscedasticity were reasonably satisfied. The assumptions of linear regression were examined through residual analysis. The normal P–P plot of standardized residuals revealed that the residuals approximately followed a diagonal line, suggesting that the normality assumption was reasonably met. The scatterplot of standardized predicted values versus standardized residuals revealed a random distribution around zero with no obvious funnel-shaped pattern, indicating homoscedasticity (constant variance of residuals).

### Multivariate linear regression analysis of factors associated with clinical nurses’ spare-time planning

Spare-time planning was designated as the dependent variable in this study. Multivariate linear regression analysis was conducted using independent variables that demonstrated significant differences in the univariate analysis, including factors such as only-child status, fertility status, professional title, nursing seniority, and frequency of weekly sports participation, number of night shifts per month, positive psychological capital, and occupational stress. The independent variables analysed are presented in Table 3. The results of the multivariate linear regression analysis indicated that the key factors associated with the shaping of clinical nurses’ spare-time planning included fertility status, frequency of sports participation each week, positive psychological capital, and levels of occupational stress. Detailed findings are presented in Table 4. The results of the multiple linear regression analysis of factors associated with the shaping of nurses’ spare-time planning are presented in Table 5.

**Table 4:** Assignment status of independent variables.

Variable name	Factors associated with	Variable assignment
X1	Only child	Yes = 1; No = 2.
X2	Fertility status	Unmarried = 1; Having one child = 2; Having two or more children = 3.
X3	Professional title	Nurse = 1; Staff Nurse = 2; Head Nurse = 3; Deputy Chief Nurse and above = 4.
X4	Length of service in nursing	Less than 5 years = 1; 5 to less than 10 years = 2; 10 to less than 15 years = 3; 15 to less than 20 years = 4; 20 years or more = 5.
X5	Frequency of participating in sports every week	0 times = 1; 1 to 3 times = 2; ≥ 4 times = 3.
X6	Number of night shift days per month	0 times = 1; 1 to 3 times = 2; ≥ 4 times = 3.
X7	Positive psychological capital	Actual value
X8	Occupational stress	Actual value
Y	Spare time planning	Actual value

**Table 5:** Multiple linear regression analysis of factors associated with planning of clinical nurses’ spare time (*n* = 827).

Independent variable	*β*	SE	*β′*	*t*	*p*	95% CI		Tol	VIF
						Lower	Upper		
Constant	17.588	2.933		5.997	<0.001	11.831	23.345		
Reproductive status	−1.843	0.792	−0.117	−2.328	0.020	−3.397	−0.289	0.373	2.678
Frequency of participating in sports activities every week	3.109	0.511	0.195	6.089	<0.001	2.107	4.112	0.915	1.093
Positive psychological capital	0.149	0.014	0.342	10.356	<0.001	0.121	0.177	0.863	1.158
Occupational stress	−0.045	0.016	−0.091	−2.748	0.006	−0.077	−0.013	0.856	1.168

The coefficient of determination is *R* = 0.488, *R*^*2*=^0.228 and the adjusted *R^2^* = 0.223; *F* = 15.840 and *p* < 0.001.

## Discussion

### Current status of spare-time planning among clinical nurses

The results indicate that the score of clinical nurses’ spare-time planning was (26.84 ± 7.80) points, reflecting a moderate level of ability to organize off-duty time. This suggests that there is a need for improvement in the spare-time planning capabilities of clinical nurses, which may be attributed to insufficient leisure guidance provided by hospital managers (16). The findings further reveal that the leisure activities engaged in by nurses predominantly consist of indoor, static, family-oriented, and labour-saving leisure activities (17). Labour-saving leisure refers to activities characterised by high passivity and low consumption of physical and mental resources when individuals pursue leisure and entertainment pursuits (18). By contrast, spare time planning involves self-directed development during off-duty hours, necessitating a high degree of initiative to pursue personal goals. Notably, entries with the highest and lowest scores aligned with the findings of Zhiqiong et al. (19). This suggests that clinical nurses tend to favour achievable goals when planning their leisure time, often beginning with self-initiated activities that enhance their capacity for self-development. However, they generally exhibit a reluctance to establish interpersonal relationships and seek challenging activities during their leisure time. Interpersonal relationships are significant sources of work-related stress for nurses, impacting both their physical and mental health. The high-intensity and high-risk nature of nursing work, coupled with difficulties in fostering positive relationships with colleagues, further constrain nurses’ abilities to expand their interpersonal networks. Additionally, the demanding nature of their clinical responsibilities often leaves nurses with insufficient time to participate in systematic training related to nursing innovation (20). This limitation hinders their capacity to engage in innovative thinking and challenging activities. Therefore, while nursing managers strive to enhance their staff’s work efficiency, they must also consider their team members’ personal lives. Encouraging participation in various leisure activities can help nurses relax and cultivate harmonious interpersonal relationships, ultimately relieving stress (21). Furthermore, the establishment of an innovative nursing training system may promote engagement in challenging recreational activities.

### Factors associated with clinical nurses’ spare-time planning

#### Fertility status

Women play a crucial role in family dynamics, and factors such as the number of family members and the presence of older adults individuals or children requiring care are significantly related to nurses’ participation in leisure activities. The findings indicate that fertility status is a key factor associated with clinical nurses’ spare time. Nurses who had not given birth exhibited the highest levels of engagement in leisure activities. This may be related to increased autonomy and availability of time for leisure pursuits, as these nurses face reduced work and family pressures. Conversely, nurses who have given birth often struggle to balance family responsibilities with their professional roles. The challenges posed by multiple children and the demands of nursing could result in limited time to engage in recreational activities. It is recommended that nursing managers actively retain nursing talent by organising shifts more effectively, providing clinical nurses with adequate time to attend to family obligations, and opportunities for spare-time planning. Additionally, relevant authorities should improve policies by providing incentives and support for family planning and fertility as well as by strengthening oversight mechanisms to ensure the establishment of childcare centres, thereby safeguarding women’s rights and facilitating the balance between childcare and professional responsibilities (22).

#### Frequency of participation in sports activities each week

The results of this study indicate that the weekly frequency of participation in sports activities is a key factor associated with nurses’ spare-time planning. Nurses who engaged in sports activities more frequently tended to plan their leisure time more effectively. Sports constitute a popular leisure activity among the public and serve as a means for individuals to relax, maintain fitness, and enjoy themselves in their free time. This represents a healthy and positive approach to entertainment and leisure (23). For nurses, participation in sports activities not only enhances their physical well-being, but also improves social communication skills and effectively alleviates work-related stress (24). Unfortunately, due to high-intensity work pressure, shift-based nursing schedules, and irregular life patterns, many nurses lack the free time and energy necessary to engage in sports activities. After work, they often prefer to rest. Research has shown (25) that some nursing staff opt for fitness, yoga, and other forms of exercise in their spare time. However, factors such as frequent plan disruptions and inadequate time management hinder their ability to maintain a consistent routine. Therefore, nursing managers should strategically allocate human resources, optimise nurses’ shift arrangements, and implement time guarantees and incentive policies to encourage participation in sports.

#### Positive psychological capital

The results indicate that positive psychological capital is positively correlated with nurses’ spare-time planning (*r* = 0.388, *p* < 0.01). Multiple linear regression analyses further revealed that positive psychological capital was a key factor associated with this variable (*p* < 0.01). Specifically, nurses with higher psychological capital scores tended to have higher levels of spare-time planning. Positive psychology, a form of beneficial psychological capital, enables nurses to effectively navigate adversity and setbacks in their work, approach life with a positive and optimistic mindset, accept their surroundings calmly, and mitigate the impact of transformation while enhancing job satisfaction (26, 27). Nurses with high levels of psychological capital tend to explore their inner potential and engage with society, thereby affirming their self-worth. This inclination may foster behaviours such as enhancing interpersonal relationships, pursuing personal development, and potentially elevating spare-time planning scores. In this positive psychological state, nurses may be better equipped to shape and plan their leisure time through constructive behaviours, potentially resulting in higher efficiency and quality of work and life (28). Consequently, it is recommended that nursing managers recognise the significance of positive psychological capital in shaping nurses’ spare-time planning abilities, actively stimulate the work enthusiasm of nursing staff, address spiritual and mental losses incurred in a complex and demanding work environment, and continually enhance nurses’ capacity to plan their leisure time.

#### Occupational stress

Occupational stress refers to the stress experienced by employees when there is a discrepancy between their career development needs and the demands of their work environment, which can disrupt their normal physiological functions (29). The results of this study indicated a significant negative correlation between occupational stress and clinical nurses’ spare-time planning(*r* = −0.234, *p* < 0.01). Furthermore, multiple linear regression analysis identified occupational stress as a key factor associated with this variable (*p* < 0.01). Specifically, lower levels of occupational stress were associated with higher levels of leisure-time planning. The findings revealed that nurses encounter a variety of stressors daily and experience relatively high levels of stress compared with other healthcare workers (30). Occupational stress may affect nurses’ spare-time planning levels differently. Research has demonstrated that elevated stress symptoms can hinder caregivers’ perceptions of motor abilities, autonomy, and sense of relevance, ultimately reducing their capacity for effective spare-time planning (31). This underscores the need for managers to recognise the role of occupational stress in relation to clinical nurses’ spare-time planning levels. Fostering a relaxed and harmonious work environment through various initiatives may effectively target and mitigate nurses’ stress, thereby promoting their physical and mental well-being.

The results of this study indicates that the shaping of clinical nurses’ leisure time was at a moderate level. Factors associated with spare-time planning include fertility status, the frequency of weekly participation in sports activities, positive psychological capital, and occupational stress. Nursing managers should develop targeted intervention measures based on these identified factors to enhance nurses’ time management skills.

## Limitations

This study has several limitations that should be acknowledged. First, the sample was recruited from four tertiary hospitals in Gansu Province, which may limit the generalizability of the findings to other regions or hospital levels. Future research should include a larger and more diverse sample from multiple regions to comprehensively examine nurses’ spare-time planning. Second, although data on both marital status and reproductive status were collected separately, these two variables were highly correlated in the present sample: all unmarried nurses had not given birth, and all married nurses had given birth. Moreover, marital status was not significantly associated with spare-time planning in the univariate analysis. Consequently, we were unable to fully extract the independent effects of reproductive status and marital status on nurses’ spare-time planning. Future studies with more diverse samples, including married, nulliparous nurses and unmarried, parous nurses, are needed to further clarify the respective roles of these two factors. Third, the data were derived from different hospitals, which may exhibit department-level clustering. Future research can consider using hierarchical linear models to account for potential clustering effects and provide more robust estimates. Additionally, it is essential to explore, formulate, and implement management plans that align with China’s cultural context to enhance nurses’ professional quality and well-being.

## Data Availability

The raw data supporting the conclusions of this article will be made available by the authors, without undue reservation.

## Footnotes

1
https://www.wjx.cn/
